# Case of Lead Palsy, Resulting from the Use of Soda-Water, Contaminated with Lead

**Published:** 1851-12

**Authors:** 


*Case of Lead Palsy, resulting from the use of Soda-Water, contaminated with Lead.* Dr. D. Macgibbon, of the Charity Hospital, N. O., reports in the N. O. Medieal and Surgical Journal, for November, 1851, a very well marked case of lead colic, followed by severe paralysis, which was attended with *blueness of the gums*—the pathognomonic sign of the presence of lead in the system—and was clearly traceable to lead taken in soda-water. "The patient kept a small store in Camp, near Julia street, in which, since the warm season commenced, she kept soda water for sale. The fountain had leaden tube attachments to it for drawing off the fluid; and on an average only one fountain of soda water was used in a week on the premises, thus leaving its contents a sufficiently long time for the free acid to act on the leaden pipe, and also for a portion of the carbonate of lead thus formed, to be diffused throughout it. She was usually in the habit, when she arose in the morning, of drawing off a glass of this beverage and drinking it; the first portion which escaped she always threw away, having heard somewhere that this was not proper to be drank. She also throughout the day would usually drink two or three more glasses of this, which was about the amount she consumed according to her own account."

Dr. M. states that "lead has, in many instances, lately been detected by the most conclusive chemical tests, in the soda water sold in N. Orleans, and that too, where less favorable circumstances prevailed than above," and he has no doubt of the saturnine origin of the case described.

